# Association of the methylation of age-related epigenetic marker ELOVL2 with neurophysiological alterations and immunosenescence during aging and its modulation by the APOE genotype

**DOI:** 10.3389/fimmu.2026.1803497

**Published:** 2026-07-14

**Authors:** Natalya V. Ponomareva, Irina L. Kuznetsova, Viktoria Petrova, Tatiana V. Andreeva, Maria S. Protasova, Rodion N. Konovalov, Marina Krotenkova, Daria D. Malina, Ekaterina Kolesnikova, Elena Kanavets, Daria Kuprianova, Andrey Mitrofanov, Julia D. Vavilova, Anna A. Boyko, Elena I. Kovalenko, Alexander M. Sapozhnikov, Vitaly F. Fokin, Sergey N. Illarioshkin, Evgeny I. Rogaev

**Affiliations:** 1 Russian Center of Neurology and Neurosciences, Moscow, Russia; 2 Vavilov Institute of General Genetics, Russian Academy of Sciences, Moscow, Russia; 3 Center for Genetics and Life Science, Sirius University of Science and Technology, Sochi, Russia; 4 Research Center of Mental Health, Moscow, Russia; 5 Shemyakin and Ovchinnikov Institute of Bioorganic Chemistry, Russian Academy of Sciences, Moscow, Russia; 6 Department of Psychiatry, Umass Chan Medical School, Shrewsbury, MA, United States

**Keywords:** aging, *APOE* genotype, *ELOVL2* methylation, event-related potentials, functional MRI, immunosenescence

## Abstract

**Background:**

DNA methylation of the *ELOVL2* (Elongation of Very Long Chain Fatty Acids Protein 2) promoter is one of the most robust molecular biomarkers for chronological age; however, whether *ELOVL2* plays a functional role in brain aging and immunosenescence, and whether the *APOE* genotype modulates these effects, has not been fully explored. This study investigated the associations among *ELOVL*2 methylation, *APOE* genotype, and neurophysiological changes in the brain, as well as indicators of immunosenescence of peripheral blood T cells during aging.

**Methods:**

We examined 72 non-demented volunteers aged 20–88 years, stratified by *APOE* genotype (41 *APOE*-, 31 *APOE*+). *ELOVL2* methylation levels were measured via bisulfite conversion and pyrosequencing. Participants underwent cognitive screening, auditory P3 event-related potential (ERP) recordings, and resting-state MRI functional connectivity (rsFC) assessments. In a subgroup of 18 healthy subjects, we examined peripheral blood T-cell subsets and analyzed the association of CD3^+^HLA-DR^+^ T cells with *ELOVL2* methylation and neurophysiological characteristics.

**Results:**

*ELOVL*2 CpG promoter methylation (chr 6:11044880. GRCh37) was associated with P3 ERP latency in both the overall sample and *APOE4*+ carriers, remaining significant even after adjusting for age; however, this association was not significant in *APOE4*- individuals. DNA methylation of *ELOVL*2 was inversely correlated with fMRI rsFC in the salience-, and memory-related, and central autonomic networks, indicating disrupted connectivity within these networks. An increased proportion of CD3^+^ cells expressing the immune activation marker HLA-DR was correlated with elevated *ELOVL*2 methylation level and with decreased fMRI rsFC in brain networks, including circuits related to the DMN medial prefrontal cortex, amygdala, and cerebellum.

**Conclusion:**

The results imply a close link between *ELOVL2* methylation, inflammaging and brain dysfunction, which is modulated by *APOE4*+ genotype.

## Introduction

Aging is a multifaceted systemic process that impacts various aspects of cellular and systemic regulation and maintenance of brain function. It is characterized by complex neural changes, often leading to declines in cognitive function. Aging is the greatest known risk factor for Alzheimer’s disease (AD) and other forms of dementia, although these disorders are not a normal part of aging.

DNA methylation, an epigenetic modification that influences gene expression, has been recognized as an important driver of biological aging (1). *ELOVL2* promoter methylation consistently increases with age across various tissues, including peripheral blood (2). As one of the most reliable epigenetic markers of chronological age, it accounts approximately for 70% of the “aging epigenetic clock” (3). Hypermethylation of the *ELOVL2* promoter is associated with reduced gene expression.

*ELOVL2* is expressed predominantly in tissues with high metabolic activity, such as the liver, retina, and brain (4). The ELOVL2 protein is an enzyme that elongates long-chain omega-3 and omega-6 polyunsaturated fatty acids (LC-PUFAs), which are precursors of 22:6n-3, docosahexaenoic acid (DHA) and very-long-chain PUFAs (VLC-PUFAs). VLC-PUFAs, particularly omega-3 fatty acids are crucial for neuronal membrane composition, synaptic function and inflammatory signaling (5, 6). Altered ELOVL2 expression driven by epigenetic modification could plausibly affect both brain physiology and systemic immune status during aging.

Age-related epigenetic changes can depend on genetic differences, such as аpolipoprotein E (*APOE*) genotype (7). The *APOE* gene has three major alleles (ϵ2, ϵ3, and ϵ4), with the ϵ4 allele being the most significant genetic risk factor for late-onset AD in Caucasian ethnic groups including the Russian ethnic population examined in this study (8–11). The glycoprotein APOE participates in lipid transport, synaptogenesis, neural repair, neuroplasticity, neuroinflammation and beta-amyloid (Aβ) clearance (12, 13, reviews). Moreover, silencing *APOE* expression in adult 5XFAD mice has been shown to reduce the amyloid burden in the brain (14).

Carriers of *APOE* ϵ4 allele (*APOE4+*) are at greater risk of age-dependent accelerated cognitive decline and neurophysiological alterations than noncarriers are (15–18). Understanding how *ELOVL2* methylation interacts with the *APOE* genotype could yield insights into how genetic and epigenetic factors jointly contribute to neural normal and pathological aging.

Neurophysiological neuroimaging techniques such as EEG, event-related potentials (ERPs), and functional MRI are valuable tools for elucidating the influence of genetic and epigenetic factors on brain function in normal aging and AD.

The oddball event-related potential (ERP) paradigm, which elicits the P3 component, has been widely used to study cognitive processing, attention, and working memory. P3 latency and amplitude are well-established neurophysiological markers of neural speed and brain efficiency. In adults, P3 latency gradually increases with age and these changes are associated with a decrease of white matter myelination (19, review; 20–23). P300 decreases in amplitude and delays in latency are related to deficits in attention and memory (24). P3 enables the detection of cognitive process abnormalities in MCI and AD patients (23, 25). Age-related alterations of ERP P3 were found to be more pronounced in carriers of AD risk genotypes (*APOE, PICALM*) (26–28). However, the influence of the *APOE* genotype on auditory ERP P3 latency in healthy adults has not been reported in several studies (29, 30), and may be modulated by other factors, including epigenetic influences.

Resting-state functional connectivity (rsFC), as measured by functional MRI (fMRI), provides insight into communication in brain networks at rest. rsFC characteristics obtained from fMRI data examine the synchronization of neural activity between regions by measuring the blood oxygen level-dependent (BOLD) signal fluctuations. The *APOE* genotype influences brain network connectivity and its age-related alterations, which may contribute to cognitive decline (31, review; 18, 32, 33).

The associations between fMRI rsFC and ERP P3, on one the hand, and characteristics and *ELOVL2* methylation, on the other hand, have not been previously investigated; however, the essential role of omega-3 PUFAs in brain membranes, synaptic function, white matter myelination, suggests that such an association may exist.

Moreover, *ELOVL*’s role in fatty acid synthesis may also interact with neuroinflammatory processes and systemic inflammation, influencing the relationships among methylation, brain function, and aging. Inflammation can disrupt normal connectivity patterns (34, review)., and methylation changes can modulate this response.

Immune senescence is characterized by a variety of markers, including a reduction in the number and function of naïve T cells, an increase in activated T cells, elevated levels of proinflammatory cytokines, and a reduced capacity for adaptive immune responses (35, 36). These changes are part of a larger phenomenon known as “inflammaging,” which describes the chronic, low-grade inflammation that accompanies aging (37). Understanding the relationship between *ELOVL2* methylation and immune senescence could shed light on the mechanisms driving these age-related changes in immune function. In particular, the CD3^+^HLA-DR^+^ T-cell phenotype reflects a state of chronic activation that is typically transient in youth but driven continuously by age-related inflammation (inflammaging) in older populations, ultimately contributing to immunosenescence (38–40). We hypothesize that increased *ELOVL2* methylation is positively associated with elevated CD3^+^HLA-DR^+^ levels, serving as a key indicator of epigenetically driven immunosenescence. Further research is also needed to better understand the relationship between peripheral markers of inflammation and neurophysiological characteristics in the aging human brain.

This study aimed to investigate the associations between *ELOVL2* methylation and characteristics of brain aging via neurophysiology (ERP P3 and fMRI rsFC of brain networks), as well as the potential influence of the *APOE* genotype on this association. This study also aimed to clarify the relationships among the immunosenescence markers in peripheral blood T cells, *ELOVL2* methylation, and these neurophysiological characteristics.

## Materials and methods

### Participants

The enrolled cohort included 72 nondemented volunteers (23 men and 49 women, aged 20–88 years). This prospective cohort study recruited community-dwelling volunteers through contact information provided by the study staff. Interested individuals underwent a clinical assessment, including a neurological examination and cognitive screening.

The subjects were of Russian descent and were from Moscow and the surrounding region. The participants underwent a neurological examination and cognitive screening. The recruited subjects were free of dementia and other medical, psychiatric, and neurological conditions. The exclusion criteria included a history of neurological and psychiatric diseases, any type of memory impairment, signs of clinical depression or anxiety, physical brain injury; other medical conditions (e.g. hypertension, diabetes, cardiac disease, or thyroid disease) or a personal history of drug or alcohol addiction.

The subjects were evaluated with the Mini-Mental State Examination (MMSE) and Clinical Dementia Rating (CDR) scale (41). Only subjects with MMSE scores of 28 or higher and CDR scores of 0 were included in the study. All the subjects were right-handed. All individuals also underwent a neuropsychological battery that included the following tests: the serial sevens subtraction test (SST) (42, 43) the controlled oral word association test (COWAT) (44), COWAT examines verbal fluency (i.e. the ability to produce words orally within a fixed time span according to phonemic constraints), a task that relies on circuits that control aspects of executive function (attention, initiation, and retrieval processes) and working memory (44).

Written informed consent was obtained from all the participants. The experimental protocol for this study was approved by the local ethics committee. *APOE* genotyping was performed for all participants, and the effects of the *APOE* genotype on neurophysiological characteristics were statistically controlled.

All subjects were divided into subgroups according to *APOE* polymorphisms. The *APOE4*+ subgroup consisted of subjects with one or more ε4 *APOE* alleles, and the *APOE4–* subgroup included subjects without any ε4 alleles.

The demographic and psychometric characteristics of the participants are presented in **Table 1**. There were no significant differences in age or psychometric characteristics between *APOE4+* carriers and noncarriers across any of the datasets, as determined by ANOVA and Mann–Whitney *U* test (categorical variables).

**Table 1:** Demographic and psychometric characteristics of the participants.

Parameter	*ALL*	*APOE4-*	*APOE4+*	*p* *APOE4- vs APOE4+*
N	72	41	31	
Age, yrs (SE)	47.9 (1.9)	45.9 (2.5)	50.5 2.9	0.2
Sex m/w	23/49	15/26	8/23	0.33
Education, yrs	15.3 (0.1)	15.2 (0.1)	15.4 (0.2)	0.5
MMSE (SE)	29.7 (0.1)	29.7 (0.1)	29.7 (0.1)	0.6
COWAT (SE)	52.2 (1.3)	51.8 (1.8)	52.8 (2.0)	0.7
SST [IQR] (%)	0.0 [0-7.1]	0.0 [0-7.1]	0.0 [0-7.1]	0.75
Errors on target [IQR] (%)	2.3 [0-2.3]	2.3 [0-2.3]	2.3 [0-2.3]	0.8

MMSE, mini-mental state examination; COWAT, controlled oral word association test; SST, serial sevens subtraction test; IQR, interquartile range.

In addition to all other examinations, fMRI rsFC was examined in a subgroup of 25 individuals, which included 7 men and 18 women (age range 29–73 years, mean age 54.1 ± 1.8). Among these individuals, 16 were *APOE4+* noncarriers (age-range 31–71 years, mean age 54.4 ± 2.0 years), and 9 were *APOE4+* carriers (age range 29–73 years, mean age 52.9 ± 5.0) (**Table 2**).

**Table 2:** Demographic characteristics of participants with fMRI data.

Parameter	*ALL*	*APOE4-*	*APOE4+*
N	25	16	9
Age, yrs (SE)	53.7 (2.3) 29-73	54.1 (2.4) 31-71	52.9 (5.0) 29-73
Sex m/w	7/18	5/11	2/7
Education, yrs	15.32 (0.90)	15.06 (0.44)	15.78 1.30
MMSE (SE)	29.68 (0.69)	29.56 (0.81)	29.89 (0.33)
COWAT (SE)	53.6 (1.9)	54.3 (2.9)	52.3 (1.8)
SST [IQR] (%)	00 [0-7.1]	0 [0-7.1]	0 [0-7.1]
Errors on target [IQR] (%)	2.3 [2.3-2.3]	2.3 [2.3-4.7]	2.3 [0-2.3]

The abbreviations are the same as those in **Table 1**.

In addition to all other examinations, we analyzed the differentiation stages and immunosenescence characteristics of T cells in a subgroup of 18 nondemented adults, which included 8 men and 10 women (age range 31–73 years, mean age 53.7 ± 2.6). Among these individuals, 12 were *APOE4+* noncarriers (age-range 31–71 years, mean age 53.5 ± 3.0), and 6 were *APOE4+* carriers (age range 33–73 years, mean age 54.0 ± 5.3 years) (**Table 3**).

**Table 3:** Demographic characteristics in a subgroup of participants who underwent immunophenotypic analysis of differentiation stages and immunosenescence characteristics of peripheral blood T cells.

Parameter	*ALL*
N	18
Age, yrs (SE)	53.7 (2.6)
Sex m/w	6/12
Education, yrs	15.1 (0.1)
MMSE (SE)	29.6 0.2
COWAT (SE)	52.6 2.6
SST [IQR] (%)	0 [0-7.1]
Errors on target [IQR] (%)	2.3 [2.3-2.3]

The abbreviations are the same as those in **Table 1**.

### Auditory ERP acquisition

Auditory ERPs were recorded according to a standard auditory discrimination protocol using target and nontarget stimuli (oddball paradigm) as previously described (27). ERPs were recorded from 17 Ag/AgCl scalp electrodes using a computerized Neuro-KM acquisition system (Statokin, Russia) via the monopolar recording with a bandpass filter of 0.16–100 Hz and a sampling rate of 512 Hz. Electrode positions were based on the International 10–20 system.

The target stimuli were recognized as 2000-Hz clicks among frequent 1000-Hz standard stimuli. The subject was required to distinguish between the two tones by mentally counting the target tones and not responding to the standard. Binaural stimuli were applied for 50 ms; the stimulus intensity was 80–90 dB (in accordance with the auditory threshold); the frequency was 1 Hz. The stimuli were applied in a pseudorandom order at a 7:3 target/nontarget ratio. Sweeps to targets were visually inspected for artefacts before being accepted into the average. The average number of target stimuli was 40-46. Control experiments demonstrated that the average number within the studied range did not influence the parameters of the cognitive ERPs. The prestimulus interval was 100 ms; the epoch length was 500 ms. We measured the N2–P3 interpeak amplitude (μV). The P3 latency was defined relative to stimulus onset. The latency and amplitude measured in the Cz area were included in further analysis.

### fMRI imaging acquisition

Structural images were acquired via a T1-weighted MPRAGE sequence: TR = 1,900 ms, TE = 2.47 ms; FOV = 256 × 256 mm2; flip angle = 10° slice thickness 1.0 mm; interslice distance 1 mm; number of slices = 176.

The following functional scans were obtained at rest using T2_-weighted EPI sequence: TR = 1,500 ms, TE = 30 ms, flip angle = 70°, slice thickness = 2 mm, FOV = 190 mm, FoV phase = 100.0%. The subjects were instructed to relax as much as possible, to lie quietly with their eyes closed (to exclude stimulation of the visual system) and not to think about anything in particular.

For the analysis of rsFC signals in the fMRI images we used CONN, which is a MATLAB-based open source toolbox (Functional Connectivity SPM Toolbox 2017, McGovern Institute for Brain Research, Massachusetts Institute of Technology) (45).

CONN toolbox version 18b in conjunction with the SPM 12 software package (Wellcome Department of Cognitive Neurology, London, United Kingdom, www.fil.ion.ucl.ac.uk/spm) was used to perform all preprocessing steps. The functional images were slice-time corrected, realigned (motion corrected), and coregistered to their respective T1-weighted anatomical images. The images were then normalized to the Montreal Neurological Institute (MNI) standard space and spatially smoothed with an 8-mm Gaussian filter.

Denoising methods was then applied to minimize the impact of artifactual sources of signal variability. This included bandpass filtering (0.01–0.1 Hz), scrubbing (volumes showing displacement larger than the 97×th percentile were censored), regressing out of the first 10 principal components (aCompCor) calculated within the maps of white matter (five components) and cerebrospinal fluid (five components) and regressing out of 24 head motion parameters, including linear and rotational indices, their temporal derivatives and their squared values.

The CONN-18.b toolbox was used to obtain a linear measure of functional connectivity based on bivariate correlation and bivariate regression coefficients between seed areas for ROI-to-ROI analysis (45). ROIs of the whole brain were drawn from the template provided by CONN (conn/rois/atlas.nii). For the purpose of the analysis, BOLD signal time courses were converted to normally distributed scores via Fisher’s transformation, which allows for the use of second-level general linear model analysis.

We performed a region of interest (ROI) analysis whereby ROIs were anatomically defined using the FSL Harvard–Oxford maximum probability cortical atlas, with bilateral regions divided into left and right hemispheres (164 ROIs).

### Methylation

The EZ Methylation Kit (Zymo Research, Irvine, CA, USA) was used to perform standard sodium bisulfite conversion on 300 ng of DNA. Bisulfite-converted samples were eluted in 20 µL of elution buffer. The concentration and quality of the converted DNA were evaluated using a NanoDrop spectrophotometer (ThermoScientific, Erlangen, DE) in the “ssDNA” mode. The absence of dsDNA in the converted sample was controlled by using the “dsDNA” mode. To amplify the target CpG (chr6:11044880, GRCh37), about 30 ng of the converted DNA was used as a template with primers published in (doi: 10.1016/j.forsciint.2020.110631; doi: 10.1016/j.fsigen.2014.10.002). To evaluate the PCR efficacy and the integrity of the bisulfite-treated DNA template, the PCR products were run on a 2% agarose gel, including a negative control and a positive control for each sample. Pyrosequencing was performed with the PyroMark 48 system (Qiagen, Hilden, Germany). The sequencing primer (doi: 10.1016/j.forsciint.2020.110631; 10.1016/j.fsigen.2014.10.002) concentration was 4 pmol. According to the guidelines, the methylation level of control cytosines (not CpG) based on pyrosequencing data was limited to 4%. The percentage DNA methylation of the target CpG was quantified via PyroMark CpG software.

### Genotyping

Genomic DNA was isolated from peripheral venous blood by the standard phenol–chloroform extraction methodology or via a Qiagen kit for DNA isolation. Genotyping was performed via PCR followed by RFLP analysis. Amplification was performed according to the manufacturer’s instructions via a Tercyc DNA amplifier (DNA-Technology, Russia) and a GeneAmp PCR System 9700 Thermal Cycler (Applied Biosystems).

To genotype the *APOE* gene locus, the following oligonucleotide primers were used: 5’_CGGCTGGGCGCG_GACATGGAGGA and 5’_TCGCGGGCCCCGGC_CTGGTACAC. The PCR protocol was as follows: preliminary denaturation at 95 °C for 4 min; 5 cycles of 95 °C for 45 s, 54 °C for 25 s, and 72 °C for 30 s; and 30 cycles of 95 °C for 5 s, 58 °C for 15 s, and 72 °C for 5 s; the last stage was performed at 72 °C for 3 min. PCR products were then cleaved by *Hha*I or *BstHH*I (SibEnzyme, Russia), and the restriction products were analyzed in a 7.5% polyacrylamide gel (46).

### Peripheral blood T cell phenotypic analysis

Venous blood samples were collected in vacuum tubes with EDTA (APEXLAB, Moscow, Russia). Peripheral blood mononuclear cells (PBMCs) were obtained by density gradient centrifugation using PolymorphPrep medium (Axis-Shield, Oslo, Norway) following the manufacturer’s instructions. After two washes (400×g, 15 min) in Dulbecco’s phosphate-buffer saline (PBS) PBMCs were stained with fluorescently labeled antibodies. The cells were incubated with antibodies for 30 min on ice in PBA staining buffer (PBS containing 0.5% bovine serum albumin (Serva, Heidelberg, Germany) and 0.01% sodium azide (AMRESCO, Inc. (VWR International, LLC), Aurora, CO, USA), washed twice in the same buffer and analyzed by flow cytometry.

The following mouse anti-human fluorescent-labeled antibodies were used: CD3-PE (Sorbent, Moscow, RU), CD3-FITC (clone FIT3a, Sony Biotechnology, San Jose, CA, USA), CD56-APC (clone N901, Beckman Coulter, Miami, FL, USA), CD4-FITC (clone RPA-T4, Sony Biotechnology, San Jose, CA, USA), CD8-PerCP (clone SK1 Sony Biotechnology, San Jose, CA, USA), aCD57-PE (clone HCD57, Sony Biotechnology, San Jose, CA, USA), HLA-DR- PE (clone L243, Sony Biotechnology, San Jose, CA, USA). The following immune cell surface markers were analyzed: CD4-FITC, and CD3-PE, and CD8-PERCP (Panel 1); CD3-FITC, and CD57-PE, and CD56-APC (Panel 2); CD3-FITC, and HLA-DR-PE, and CD56-APC (Panel 3). Flow cytometry analysis was carried out on a FACSCalibur flow cytometer (BD Biosciences, Franklin Lakes, NJ, USA) equipped with 488 and 640 nm lasers and an appropriate set of detectors and filters. The data were processed via FlowJo X 10.0.7r2 (FlowJo LLC, Ashland, OR, USA).

Flow cytometric data: gating strategy and representative histograms are shown in **Supplementary Figure 1**.

### Statistical analysis

#### Demographic and clinical characteristics

Differences in demographic scores between the groups (*APOE4-*, *APOE4+*), were tested via analysis of variance (ANOVA) for continuous variables and the Mann–Whitney *U* test for categorical variables and non-normally distributed data.

#### Normality testing and data transformation

The ERP P3 latency and amplitude from each group were tested for normality via the Kolmogorov-Smirnov test, and in all cases, the data were found to be normally distributed.

The normality of the CpG *ELOVL2* methylation values was assessed via the Kolmogorov-Smirnov test, which indicated a non-normal distribution (*p < 0.05*). To address this, a natural log-transformation was applied to normalize the distribution. Following the transformation, the Kolmogorov-Smirnov test confirmed that the log-transformed *ELOVL2* methylation values followed a normal distribution.

Similarly, the Kolmogorov-Smirnov test was used to evaluate the normality of T-cell subset values, which indicated a non-normal distribution (*p < 0.05*). To address this, a logit transformation was applied to normalize the distribution of CD3^+^HLA-DR^+^ T cells (17). The logit-transformed CD3^+^HLA-DR^+^ values were normally distributed.

#### Correlation

Pearson correlation analyses were conducted to examine the relationships between ERP parameters and age, between log-transformed *ELOVL2* methylation values and age, and between ERP P3 parameters and log-transformed *ELOVL2* methylation values.

#### Regression modeling, analysis of variance

Separate general linear model (GLM) regression analyses were conducted using *ELOVL2* methylation as the predictor variable, with ERP P3 latency and amplitude as the dependent variables. These characteristics were analyzed separately because P3 latency and amplitude reflect distinct underlying neural processes – specifically, processing speed versus cognitive resource allocation. To control for potential confounding factors, chronological age and sex were included as covariates in both models.

Additionally, a two-way ANCOVA was performed to assess the effects of the *APOE4* genotype and log-transformed *ELOVL2* methylation levels (categorized as higher vs. lower than the mean) on P3 latency, while controlling for age and sex.

Association between *ELOVL2* methylation and the CD3^+^HLA-DR^+^ subset was examined via GLM regression analysis using *ELOVL2* methylation as the predictor variable, logit-transformed CD3^+^HLA-DR^+^ cell levels as dependent variable, chronological age and sex as covariates.

#### Analysis of fMRI functional connectivity

Regression analyses between fMRI ROI-ROI connectivity strength and age, *ELOVL2* methylation, and the CD3^+^HLA-DR^+^ subset were conducted directly in the Conn Toolbox via generalized psychophysiological interaction (gPPI) analysis (45). Association of fMRI rsFC with both *ELOVL2* methylation and the CD3^+^HLA-DR^+^ subset was assessed, controlling for chronological age as a covariate. The results were evaluated using an analysis-level False Discovery Rate (FDR) correction (*p < 0.05*). This correction takes into account the total number of pairwise correlations run in our ROI-ROI analysis (164 х163/2) and computes the FDR correction accordingly.

## Results

### Correlation of epigenetic and neurophysiological characteristics with chronological age in nondemented adults

An assessment of the bivariate relationships of *ELOVL2* methylation and ERP with chronological age was conducted to establish their dependence within the sample prior to the subsequent multivariate regression analyses. In the examined sample of nondemented adults, methylation of the *ELOVL2* CpG site was highly correlated with age (r=0.58, *p* = 0.0000001) (**Supplementary Figure S1A**). The correlation was robust in both *APOE4-* (r=0.54, *p* = 0.0003) and *APOE4+* carriers (r=0.61, *p* = 0.0003), confirming the consistency of this association (**Supplementary Figure S1B****).**

ERP P3 latency was positively correlated with chronological age (r=0.58, *p* = 0.0000001) (**Supplementary Figure S2A**). The correlation between P3 latency and chronological age was significant in *APOE4-* carriers (r=0.66, *p* = 0.000003) as well as in *APOE4+* carriers (r=0.56, *p* = 0.001), yielding a robust association in both groups (**Supplementary Figure S2B**).

Chronological age did not significantly correlate with ERP P3 amplitude in the entire sample (r=-0.02, *p* = 0.9), the *APOE4*+ carriers (r = -0.10, *p* = 0.6) or noncarriers (r =0.07, *p* = 0.7).

### Association of ELOVL2-methylation and ERP P3 characteristics

**Figure 1:**
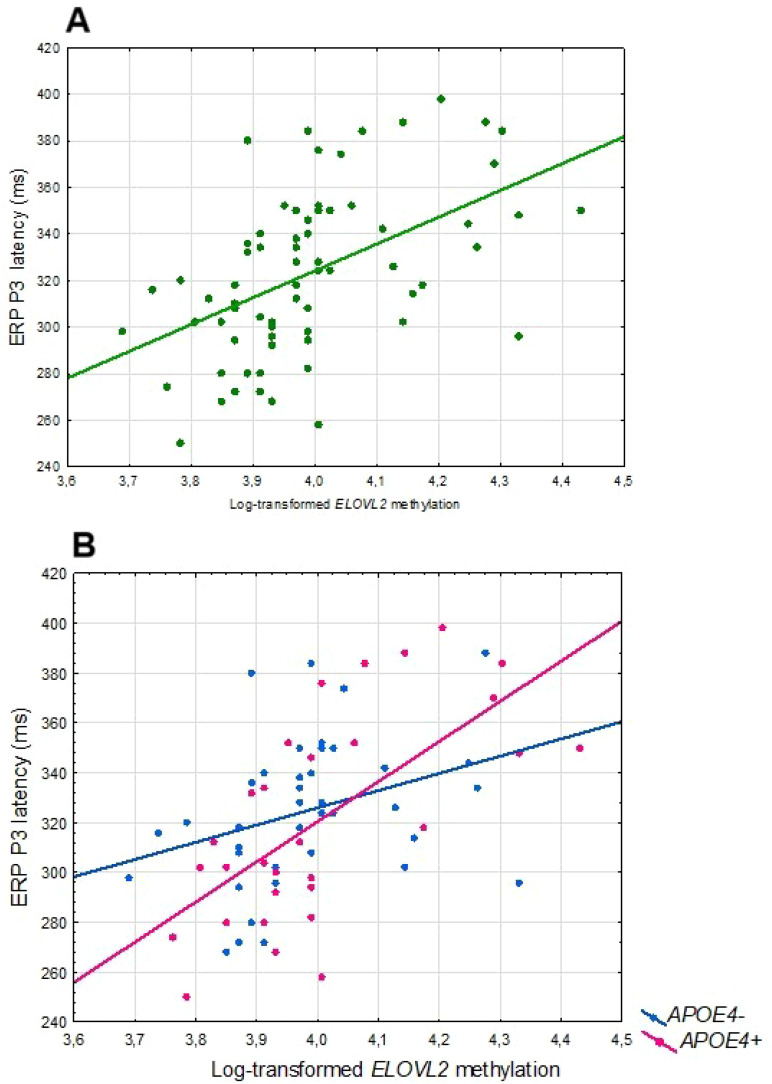
Association between *ELOVL2* CpG methylation and ERP P3 latency in the nondemented adults (entire sample) **(A)**, and in carriers and noncarriers of the *APOE4+* genotype **(B)**.

A GLM linear regression analysis revealed a significant association between *ELOVL2* methylation and ERP P3 latency, even after adjusting for the effects of age and sex. This association was present both within *APOE4*+ carriers and across the entire sample, though the effect size was weaker in the combined group (**Figure 1**; **Table 4**). The overall model was statistically significant and accounted for a substantial proportion of the variance in P3 latency (**Table 5**). Specifically, the semi-partial correlation (*sr*) for the main predictor, *ELOVL2* methylation, was significant both in *APOE4+* carriers (*sr* = 0.41, *p* = 0.005) and in the combined group (*sr* = 0.21, p = 0.03). Among the controlled covariates, age was significantly associated with P3 latency in both groups, whereas sex had no significant effect.

**Table 4:** Results of linear regression analysis evaluating the association between *ELOVL2* methylation and ERP P3 latency in non-demented adults, stratified by *APOE* genotype.

Parameter	All subjects	*APOE4-*	*APOE4+*
*ELOVL2* methylation *beta (SE*) [95%CI]	0.26* (0.12) [0.03, 0.49]	-0.02 (0.15) [-0.33, 0.28]	0.52** (0.17) [0.17, 0.88]
Age *beta (SE*) [95%CI]	0.43** (0.12) [0.20, 0.66]	0.67**** (0.15) [0.37, 0.97]	0.19 (0.18) [-0.18, 0.56]
Sex *beta (SE)* [95%CI]	0.03 (0.10) [0.03, 0.49]	0.02 (0.13) [-0.24, 0.27]	-0.16 (0.14) [-0.46, 0.13]

*ELOVL2* methylation values were log-transformed prior to analysis Data are presented as regression *beta* coefficients with standard errors (*SE*) and 95% confidence intervals [95%CI].

*p < 0.05, **p < 0.01, ***p < 0.001, ****p < 0.00001.

**Table 5:** Model summary of the linear regression analysis of association between *ELOVL2* methylation and ERP P3 latency in non-demented subjects, stratified by *APOE* genotype.

Parameter	All subjects	APOE4-	APOE4+
Multiple R	0.62	0.66	0.71
Multiple R2	0.39	0.43	0.50
Adjusted R2	0.36	0.38	0.45
F (df)	14.34 (3, 68)	9.34 (3, 37)	9.06 (3,27)
P	0.0000002	0.0001	0.0003

Conversely, among *APOE4* noncarriers, the association between *ELOVL2* methylation and P3 latency was not significant, although the overall model remained significant (**Tables 4**, **5**). The age covariate accounted for the majority of the explained variance, and the semi-partial correlation for the main predictor, *ELOVL2* methylation, was negligible (*sr =* -0.02, *p* = 0.87).

These results suggest a link between increased *ELOVL2* methylation and prolonged P3 latency in the entire sample and in the *APOE4+* carriers specifically; however, no significant association was observed in *APOE4-* individuals.

**Figure 2:**
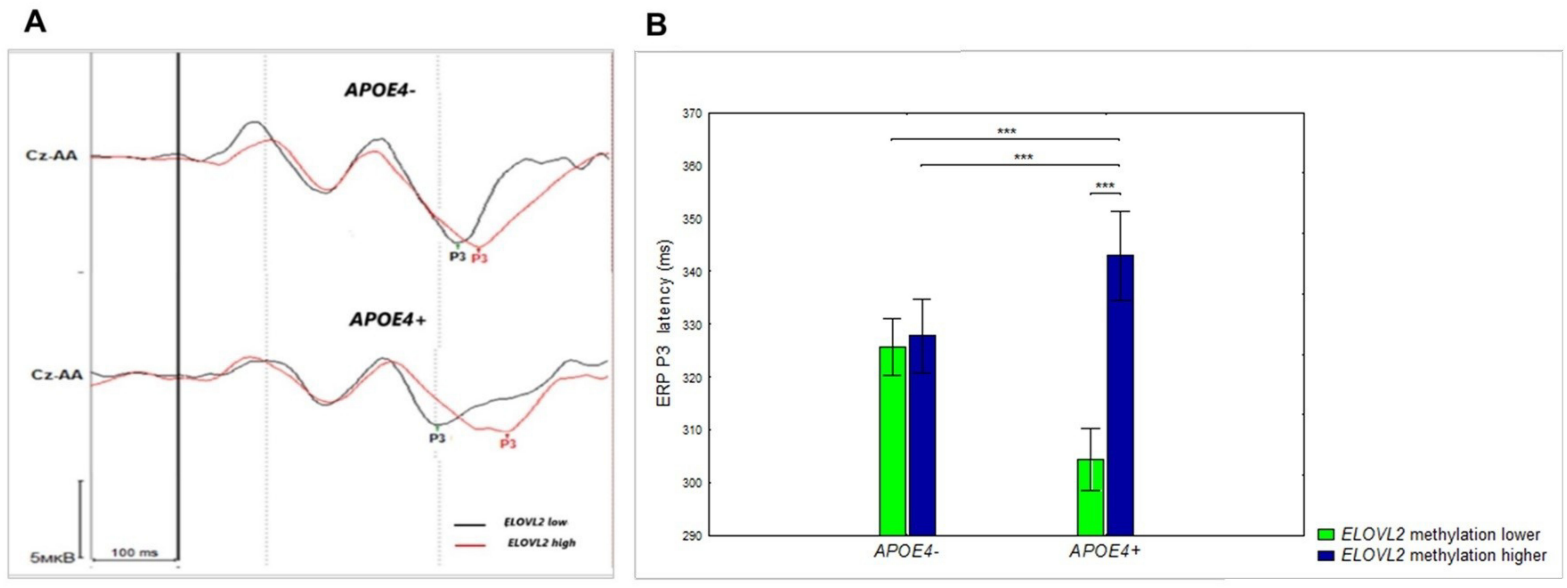
Averaged ERPs to the target tone in *APOE4+* carriers and noncarriers with low (below the mean) and high (above the mean) *ELOVL2* methylation levels. **(A)** ERP waveforms; **(B)** mean ERP P3 latency. 1, low, 2, high *ELOVL2* methylation levels. *** p < 0.001.

Additionally, a two-way ANCOVA was performed to assess the effects of the *APOE4* genotype and log-transformed *ELOVL2* methylation levels (categorized as higher vs. lower than the mean) on P3 latency, while controlling for age and sex revealed a significant main effect of both log-transformed *ELOVL2* methylation and *APOE* genotype on P3 latency (*F*(1, 67) = 7.98, *p* = 0.006). Bonferroni-corrected *post-hoc* analyses indicated that, among *APOE4+* carriers, prolonged P3 latency was strongly associated with higher *ELOVL2* methylation levels (*p* < 0.0001), whereas this association was not significant in *APOE4-* individuals (*p* = 0.2). Furthermore, within the higher *ELOVL2* methylation subgroup, *APOE4+* carriers exhibited significantly longer P3 latencies compared to *APOE4* non-carriers (*p* = 0.0009) (**Figure 2**). These ANCOVA results also demonstrated an *APOE*-dependent association between *ELOVL2* methylation and ERP P3 latency. A linear regression analysis showed no significant association between *ELOVL2* methylation and ERP P3 amplitude in any group (p > 0.05).

### Association of fMRI resting-state functional connectivity with chronological age and CpG in the ELOVL2 promoter methylation in in nondemented individuals

fMRI rsFC analysis enabled a more detailed examination of brain networks associated with *CpG* in the *ELOVL2* promoter methylation. Because of the limited number of individuals with both fMRI and CpG in the *ELOVL2* methylation dataset available (16 *APOE4-* and 9 *APOE4+* carriers), an association analysis was conducted on the sample, including both *APOE4+* and *APOE4-* carriers.

In the overall sample increased age was associated with decreased connectivity in several brain circuits (**Supplementary Figure 3**; **Supplementary Table 1**). including interhemispheric salience-related networks, dorsal attention- and visual-related networks, and cerebellum-related networks. However, older age was associated with increased rsFC in the circuit between the right amygdala and the right supramarginal gyrus. The data indicate age-dependent functional connectivity alterations within major large-scale brain networks.

**Figure 3:**
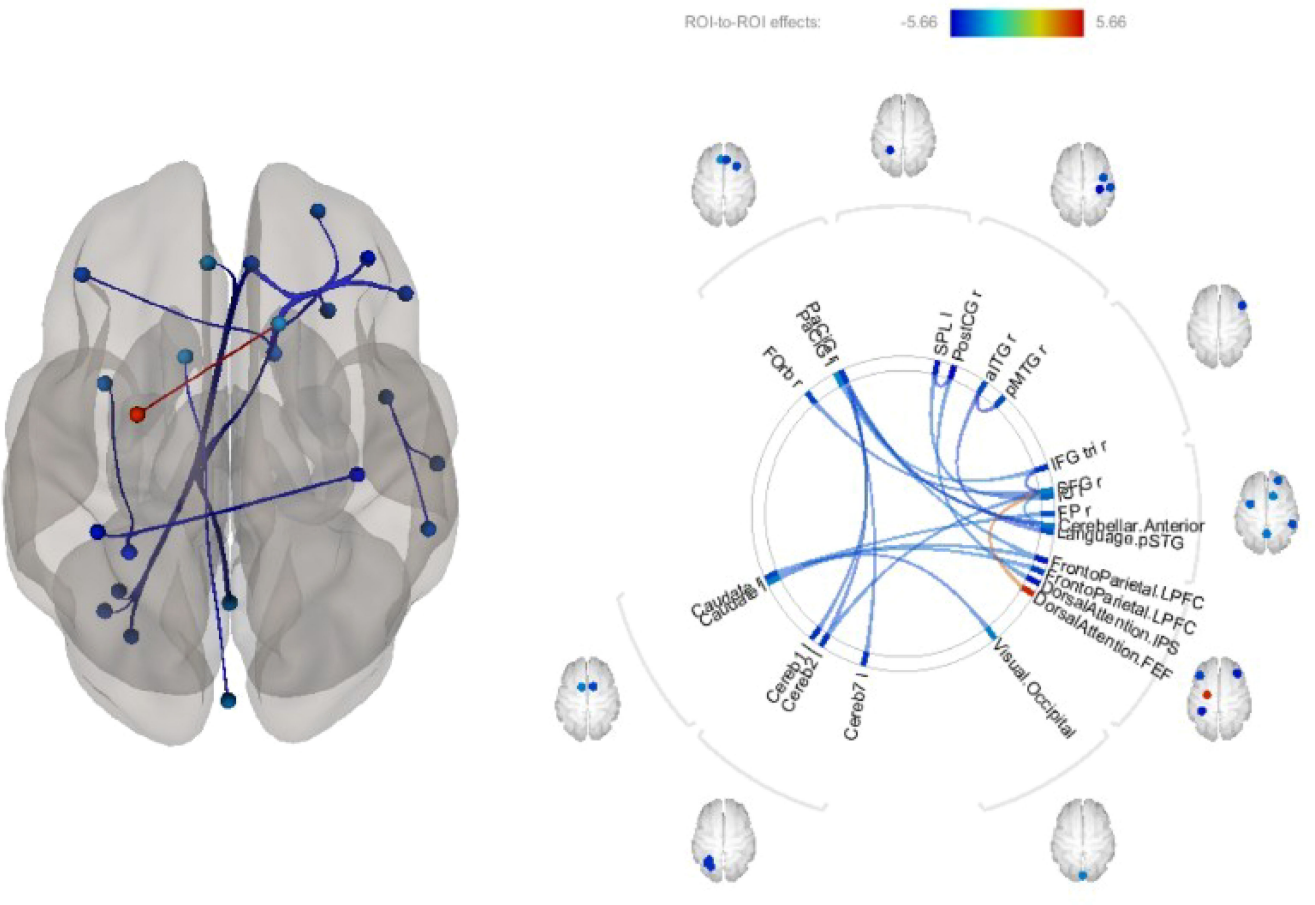
Patterns of circuits in which fMRI resting-state functional connectivity (rsFC) is associated with *ELOVL2* CpG methylation (higher vs. lower *ELOVL2 CpG* methylation relative to the mean log-transformed *ELOVL2* methylation value) among nondemented adults. Abbreviations are the same as those in **Table 6**.

Association of fMRI rsFC with *ELOVL2* methylation was examined, controlling for chronological age as a covariate. *ELOVL2* CpG promoter methylation was associated with reduced rsFC across the dorsal attention, salience, cerebellar, caudato-frontal, caudato-occipital, and insulo-parietal networks (**Figure 3**; **Table 6**). An increase in connectivity associated with *ELOVL2* CpG methylation was observed only for the rsFC between the right superior frontal gyrus and the left frontal eye field. The results suggest a significant association between *ELOVL2* methylation and functional connectivity alterations in essential large-scale brain networks, even after controlling for age.

**Table 6:** ROI-to-ROI fMRI resting-state functional connectivity (rsFC) associated with *ELOVL2* CpG methylation (higher vs. lower *ELOVL2 CpG* methylation relative to the mean log-transformed *ELOVL2* methylation value) in nondemented adults adjusted for chronological age.

Analysis Unit	T	*p-unc*	*p-FDR*
pMTG r - aITG r	-5.66	0.0000	0.0018
SFG r - IFG tri r	-5.19	0.0000	0.0055
aITG r - Network Language pSTG R	-4.79	0.0001	0.0071
SPL l -PostCG r	-5.04	0.0000	0.0078
PaCiG r - Network Cerebellar Anterior	-4.72	0.0001	0.0104
PaCiG r -Cereb2 l	-4.50	0.0002	0.0104
PaCiG r -Cereb1 l	-4.41	0.0002	0.0104
PaCiG r -Cereb7 l	-4.35	0.0003	0.0104
SFG r -FOrb r	-4.48	0.0002	0.0152
SFG r -Cereb2 l	-4.20	0.0004	0.0202
PaCiG r -Network FrontoParietal LPFC R	-3.96	0.0007	0.0215
PaCiG r -IFG tri r	-3.88	0.0008	0.0221
SPL l - IC l	-4.21	0.0004	0.0292
FOrb r - SFG r	-4.48	0.0002	0.0305
SFG r - Network DorsalAttention FEF L	3.82	0.0009	0.0321
SFG r - Network Cerebellar Anterior	-3.80	0.0010	0.0321
PostCG r - Network DorsalAttention IPS L	-4.16	0.0004	0.0336
Caudate l-Network VisualOccipital	-4.41	0.0002	0.0360
Network Cerebellar Anterior - PaCiG l	-4.08	0.0005	0.0402
Caudate r - FP r	-4.09	0.0005	0.0465
Caudate r - Network FrontoParietal LPFC R	-4.00	0.0006	0.0465
Caudate r- Network FrontoParietal LPFC L	-3.86	0.0009	0.0465

Abbreviations are the same as those in **Figure 3**.

Higher and lower *ELOVL2* CpG methylation groups were determined relative to the sample mean of the log-transformed methylation values. p-unc, uncorrected p-values; p-FDR, false discovery rate-adjusted p-values;Abbreviations: MTG, middle temporal gyrus; aITG, inferior temporal gyrus anterior division; Network Language pSTG, language network of superior temporal gyrus, posterior division; SPL, superior parietal lobule; PostCG, postcentral gyrus; PaCiG, paracingulate gyrus; Cereb2, cerebellum 2^nd^ division; SFG, superior frontal gyrus; Network DorsalAttention FEF, dorsal attention network frontal eye field, FOrb, frontal orbital cortex; IC, insular cortex; Network DorsalAttention IPS, dorsal attention network intraparietal sulcus; Network FrontoParietal LPFC, frontoparietal network lateral prefrontal cortex; Caudate, caudatum; FP, frontal pole; IFG tri, inferior frontal gyrus, pars triangularis; Network VisualOccipital – visual occipital network; other.

### Associations between systemic markers of inflammaging, ELOVL2 methylation and brain dysfunction

The subsets of T cells in the examined cohort are presented in **Table 7**.

**Table 7:** Proportion of T cells (CD3^+^), including CD4^+^ and CD8^+^ subsets, and CD57^+^ in nondemented adults.

Subset	Gate	% of lymphocytes	N
CD3^+^	Lymphocytes	51.8 ± 3.5	18
CD4^+^CD8^–^	CD3^+^	64.6 ± 3.1	17
CD4^–^CD8^+^	CD3^+^	25.6 ± 2.6	17
CD3^–^CD56^+^	Lymphocytes	13.3 ± 1.7	18
CD57^+^	CD3^+^CD56^–^	11.6 ± 1.9	18
CD57^+^	CD3^–^CD56^+^	54.0 + 4.2	18
CD3^+^HLA-DR^+^	Lymphocytes	3.8 ± 0.4	18

**Figure 4:**
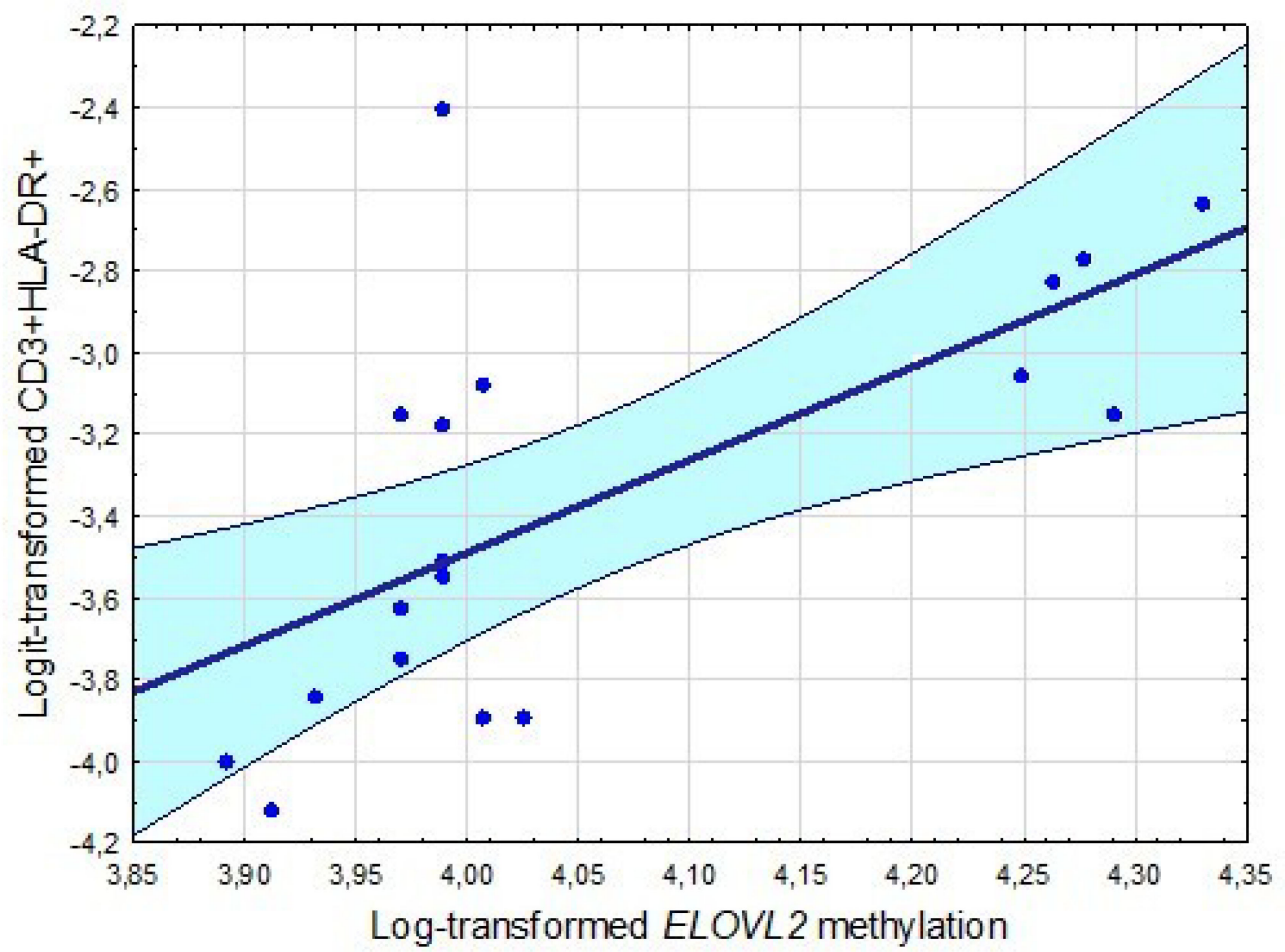
Association between *ELOVL2 CpG* methylation and logit-transformed CD3^+^HLA-DR^+^ T cells levels in nondemented adults.

A GLM regression analysis, adjusted for chronological age and sex, demonstrated a significant association between *ELOVL2* methylation and logit-transformed CD3^+^HLA-DR^+^ proportions (*R*2 = 0.48, *adj. R2* = 0.36, F(3, 14) = 4.20, p = 0.03) **Figure 4**. As shown in **Table 8**, *ELOVL2*-methylation was a significant positive predictor of the CD3^+^HLA-DR^+^ cell levels, suggesting a link between this epigenetic aging marker and heightened T-cell activation. The semi-partial correlation for the main predictor, *ELOVL2* methylation, was significant (*sr* = 0.59, *p* = 0.009). Observed power was 0.81 (at α=0.05), meeting the standard 0.80 threshold for adequate statistical power.

**Table 8:** Results of linear regression analysis of the association between *ELOVL2* methylation and CD3^+^HLA-DR^+^ cell levels in non-demented adults.

Variable	*Beta*	*beta SE*	-95% CI	+95% CI
*ELOVL2* methylation	0.60	0.20	0.18	1.03
Age	0.23	0.20	-0.21	0.67
Sex	0.02	0.20	-0.41	0.45

A logit transformation was applied to CD3^+^HLA-DR^+^ cell levels. Other abbreviations remain consistent with **Table 4**.

**Figure 5:**
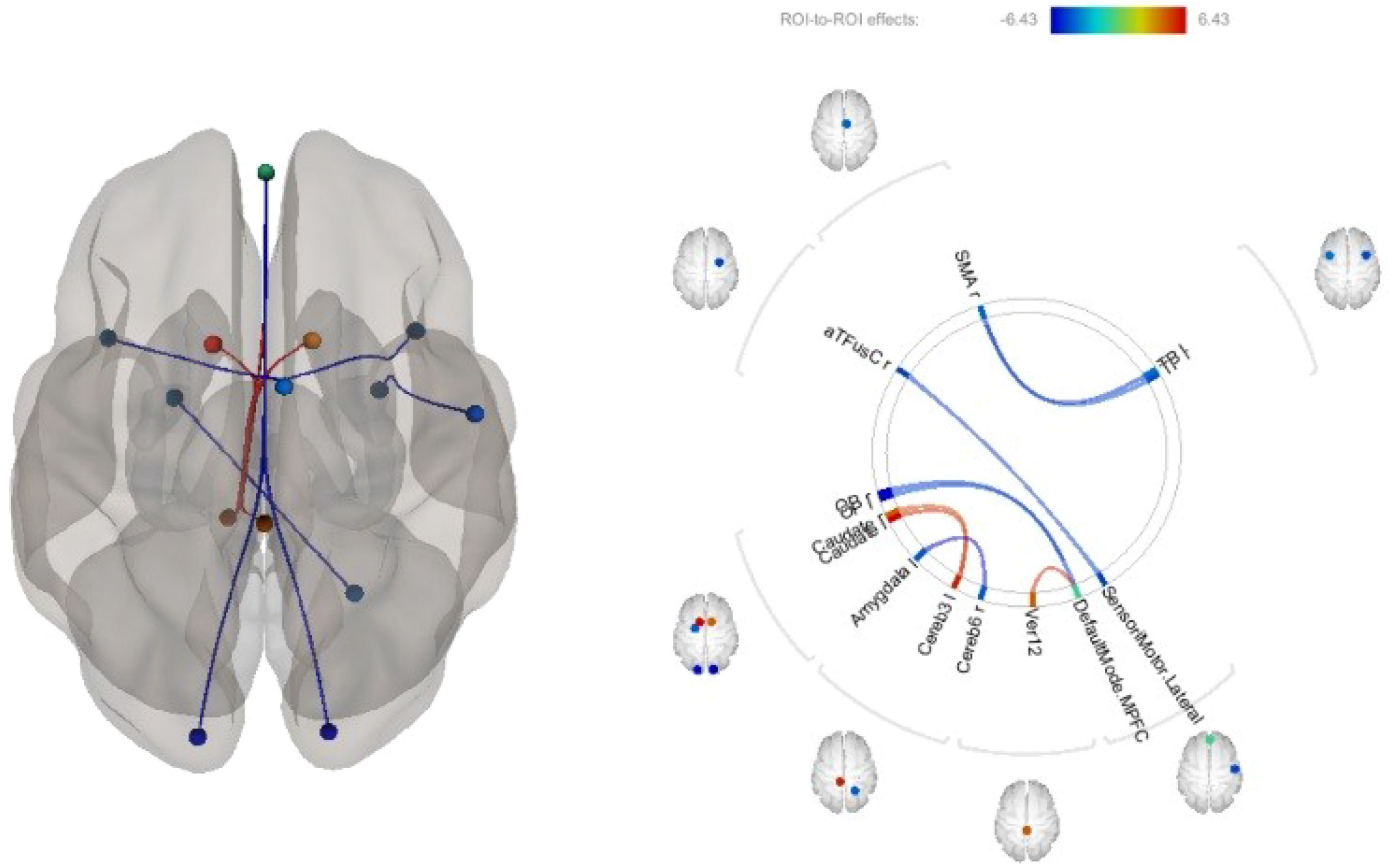
The pattern of circuits in which fMRI resting-state functional connectivity (rsFC) is associated with an increase in CD3^+^HLA-DR^+^ T cells in peripheral blood in nondemented individuals. The results of regression analysis. The abbreviations are the same as in **Table 9**. The orange lines represent positive associations, and the blue lines represent negative associations.

To assess whether an increase in activated T cells in peripheral blood is related to brain functional activity, we examined the associations of CD3^+^HLA-DR^+^ T cells with fMRI rsFC characteristics. The results revealed that an increase in CD3^+^HLA-DR^+^ T cells was associated with reduced rsFC in the MPFC- and amygdala-related networks, in cerebellar circuits and in interhemispheric network between SMA r (right supplementary motor cortex) and TP l (left temporal cortex). Functional connectivity in caudate-cerebellar circuits was positively associated with level of CD3^+^HLA-DR^+^ T cells (**Figure 5**; **Table 9**). These data indicate that elevated levels of activated CD3^+^HLA-DR^+^ in the peripheral blood are associated with neural dysregulation, characterized by rsFC deficits in cortical and amygdala networks alongside localized hyperconnectivity within caudate-cerebellar circuits.

**Table 9:** ROI-to-ROI fMRI resting-state functional connectivity (rsFC) associated with an increase of CD3^+^HLA-DR^+^ T cells in peripheral blood in nondemented individuals.

Analysis unit	*T*	*p-unc*	*p-FDR*
Amygdala l-Cereb6 r	-6.43	0.0000	0.0037
Caudate l-Cereb3 l	5.72	0.0001	0.0115
Ver12 - Network DefaultMode MPFC	5.70	0.0001	0.0119
Network DefaultMode MPFC - OP r	-5.10	0.0002	0.0147
Network DefaultMode MPFC-OP l	-4.94	0.0003	0.0147
TP l -SMA r	-5.43	0.0001	0.0189
Cereb3 l -Caudate r	4.92	0.0003	0.0228
aTFusC r - Network SensoriMotor.Lateral R	-5.21	0.0002	0.0273
SMA r -TP r	-4.78	0.0004	0.0295

The results of regression analysis, adjusted for chronological age. Abbreviations are the same as in **Figure 5**.

Network DefaultMode MPFC, default mode network medial prefrontal cortex; OP, occipital pole; OFusG, occipital fusiform gyrus; SMA, supplementary motor area; TP, temporal pole, other abbreviations remain consistent with **Table 6**.

## Discussion

The main findings of our study demonstrated an association between CpG in the *ELOVL*2 promoter methylation and P3 ERP latency. This association remained significant even after adjusting for age and sex, despite both *ELOVL2* methylation and ERP latency being correlated with chronological age. Notably, the correlation between CpG in the *ELOVL2* methylation regions and ERP P300 latency was stronger in *APOE4*+ carriers than in noncarriers and remained significant for carriers even after controlling for age.

Further analysis allowed us to identify brain networks where reduced fMRI functional connectivity was linked to DNA methylation at the *ELOVL2* promoter. This included reduced rsFC across the dorsal attention, salience, and cerebellar networks. Moreover, *ELOVL2* methylation correlated with a marker of immune activation — an increased proportion of CD3^+^HLA-DR^+^ cells in peripheral blood. CD3^+^HLA-DR^+^ also correlated with decreased fMRI rsFC in brain networks, including circuits related to the DMN medial prefrontal cortex, amygdala, and cerebellum.

### Implication of ELOVL2 methylation in aging

Our findings of a positive correlation between CpG methylation in the *ELOVL2* promoter and age align with those reported in previous studies (47–49 for review). Age-methylated CpGs, including those in *ELOVL2*, are enriched at targets of Polycomb Repressive Complex 2 (PRC2), which represses gene expression through histone H3K27 methylation and chromatin compaction to regulate aging (50, 51). Hypermethylation of the *ELOVL2* promoter is associated with reduced gene expression, leading to decreased production of omega-3 PUFAs (3, 52, 53).

Alterations in lipid metabolism play a causative role in age-related diseases, including those associated with cognitive decline, retinal degeneration, and glucose intolerance (3, 4, 54–56). Omega-3 PUFAs influence blood–brain barrier (BBB) integrity and glymphatic function (57). There is direct evidence of an association between p-tau protein burden in the hippocampus — a hallmark of AD — and the methylation level of the *ELOVL2* gene (58). Additionally, these authors reported *ELOVL2* hypermethylation in early-stage AD patients compared with controls. Omega-3 PUFAs may lower the production and increase clearance of amyloid-beta (3, 59, 60).

Moreover, the *ELOVL2* gene, which affects omega-3 PUFA levels, plays a role in the innate immune defense system and inflammatory processes, particularly in macrophage function (37, review; 5).

### P3 ERP latency as a marker of cognitive aging and its relationship with ELOVL2 methylation and the APOE genotype

The P3 component, particularly observed in oddball paradigms, is one of the most studied ERPs in relation to cognitive processes such as attention and memory. Our results showing an increase in P3 latency with chronological age are consistent with previous studies, which indicated that P3 latency typically elongates with age, and delayed P3 latency is associated with attention and memory deficits (19, review; 20–24). These findings suggest that P3 latency may serve as a potential marker for cognitive aging. The decrease in neural processing efficiency is attributed to several factors such as reduced myelination, neuronal loss, and synaptic dysfunction (19, 61, review).

The relationship between *ELOVL2* methylation and P3 latency has not been previously investigated. However, the positive effect of very-long-chain PUFA administration on P300 latency in elderly men has been previously reported (62). Taken together, these data and our results suggest that the influence of *ELOVL2* hypermethylation on brain function may be mediated through moderating effect on PUFA synthesis.

VLCFAs play important roles in membrane stabilization, synaptic function, and neuronal myelination. ELOVL2 is localized in the endoplasmic reticulum (ER), where PUFAs are elongated. ELOVL2 insufficiency may lead to chronic ER stress, resulting in the accumulation of misfolded proteins and mitochondrial dysfunction, which causes oxidative stress (3, review). VLCFAs play important roles in membrane stabilization, synaptic function, and neuronal myelination and maintaining white matter integrity as well as in signaling pathways (63, 64). All of these factors may contribute to reduced brain integrity, which underlies prolonged P3 latency. As VLCFAs levels were not measured in the present study, the proposed link remains hypothetical and requires further investigation.

Our results regarding the influence of the *APOE* genotype on the association between *ELOVL2* methylation and P3 latency may be due to the synergistic effects of the *APOE4+* genotype and *ELOVL2* hypermethylation on brain integrity in aging.

### Synergistic negative effects of the APOE4+ genotype and increased ELOVL2 methylation on ERP P3 latency during aging

*APOE* genotype influences lipid metabolism in the brain and peripheral tissues, leading to reduced DHA delivery to the brain even before dementia begins (65–67). In *APOE4+* carriers, the impact of *ELOVL2* methylation on fatty acid availability may be amplified, leading to more pronounced impairments in neuroplasticity, synaptic function, white matter myelination, energy metabolism and neuroinflammation (12, 13, reviews; 68). Poor lipid profiles could exacerbate the effects of *ELOVL2* downregulation, affecting neural efficiency. *APOE* has an impact on the brain amyloid burden (14).

Moreover, the *APOE4+* genotype is directly associated with increased accumulation of amyloid plaques and tau tangles in the brain (69, review). *ELOVL2* hypermethylation may further exacerbate these processes by impairing PUFAs metabolism. There is a substantial body of evidence indicating a direct link between PUFAs and the processing of amyloid in the brain (70)

Changes in lipid metabolism due to *ELOVL2* alterations, combined with the impacts of *APOE*, may disrupt neural communication, further contributing to delayed P3 responses. The interaction between *ELOVL2* methylation and *APOE* genotype may also affect the neural circuitry underlying cognitive tasks.

Our *APOE* stratification results indicate neurobiological complexity underlying the *ELOVL2* methylation association. Notably, association between *ELOVL2* methylation and P3 latency remains robust only in *APOE4+* carriers, vanishing in the *APOE4-* cohort. This divergence indicates that the relationship between *ELOVL2* methylation and functional electrophysiological changes is genotype-contingent, rather than simply moderated by *APOE* status. This suggests that the functional impact of *ELOVL2* dysregulation on neural processing speed may only manifest, or become accelerated, under the specific metabolic or neurodegenerative vulnerabilities conferred by the *APOE4* allele.

These results suggest that *ELOVL2* methylation has the potential to become a valuable biomarker specifically within *APOE4+* cohorts.

### Interactions of ELOVL2 CpG methylation with fMRI rsFC networks in aging

An fMRI rsFC analysis was conducted to obtain a more detailed examination of brain networks associated with *ELOVL2* CpG methylation. Previous resting-state fMRI studies have consistently demonstrated that the integrity of brain networks decreases with age. In aging, within networks fMRI rsFC typically decreases, while rsFC between networks may increase, resulting in between-network dedifferentiation (71–74). Reduced rsFCs result from complex alterations of white matter microstructural integrity, gray matter atrophy, synaptic dysfunction, and decreased metabolic activity (75, 76). Disruption of brain networks rsFC with aging could potentially mediate changes in cognitive performance (77). More pronounced progressive disruption of brain networks connectivity was found in MCI and AD, which can be characterized as a disconnection syndrome (78).

Our data are in agreement with previous results. In the overall sample, increased chronological age was associated with reduced connectivity in several brain circuits, including interhemispheric salience-related networks, dorsal attention and visual networks, cortical-subcortical circuits involving cerebellum-related networks. Increased connectivity was found in the right amygdala–SMG circuit. This increase may be compensatory but may also contribute to heightened sensitivity to negative emotional stimuli and stress (79).

The results of the present study revealed that the patterns of disrupted connectivity associated with *ELOVL2* CpG methylation and chronological age were not identical, however, in both cases reduced rsFC was observed across dorsal attention- and salience-related circuits, and in cerebellar networks, suggesting disrupted connectivity among these networks. We also found that *ELOVL2* CpG methylation was associated with reduced rsFC in insulo-parietal networks. The insular cortex plays a crucial role in the autonomic nervous system, and disruption of insulo-parietal circuits may be involved in the dysregulation of these functions in individuals with *ELOVL2* CpG hypermethylation. Disruption of insula-related networks may contribute to the AD development, as the insular cortex is affected in its preclinical stages (80, review).

Our data indicate that *ELOVL2 CpG* methylation and aging were associated with reduced connectivity of brain structures across dorsal attention networks. Previous studies have demonstrated the involvement of dorsal attention circuits in performance on oddball tasks (81, 82, review). Therefore, the disruption of the dorsal attention networks observed in the present study, may underlie the prolonged P3 latency in aging and increased *ELOVL2* CpG methylation.

The only increased connectivity associated with *ELOVL2* CpG hypermethylation was in the circuit related to the superior frontal gyrus. This increase may serve as a compensatory mechanism to counterbalance age-related declines in attention and cognitive control.

We did not find studies on the association between fMRI rsFC and *ELOVL2* methylation, but the results of research on the association between serum omega-3 PUFAs or prior omega-3 intake and brain MRI indirectly corroborate our findings. Decreased omega-3 PUFAs in blood were associated with reduced total brain white matter volume (83), as well as with decreased functional connectivity within regions that support executive function (prefrontal cortex), memory (hippocampus), and emotion (amygdala) (84). Omega-3 PUFA supplement intake reduced neuronal integrity breakdown in *APOE4+* carriers, suggesting that this treatment may be beneficial for this specific group (85).

### Associations between systemic markers of inflammaging, ELOVL2 methylation and brain dysfunction

The results of the present study showed that *ELOVL2* CpG methylation was associated with an increased proportion in peripheral blood of the T cells expressing HLA-DR, a marker of T cell activation (86). An age-related increase in the CD3^+^HLA-DR^+^ cells subset has been previously observed in several studies (38, 87). The age-dependent elevation of HLA-DR^+^ memory T helper cells was revealed during single-cell profiling of peripheral blood mononuclear leukocytes (36). The age-associated shifts in the T cell subset structure, namely, the increased proportion of activated T cells, may reflect chronic inflammation and a dysregulated immune system, both common in older adults — a process known as inflammaging (37). In the present study, the association of CD3^+^HLA-DR^+^ T cells with *ELOVL2* methylation was significant even adjusting for age and sex. This immune marker was not significantly associated with chronological age, which may be related to several factors, including a small sample size.

An imbalance in PUFAs caused by increased *ELOVL2* methylation may promote a proinflammatory state, T-cell activation and proinflammatory cytokine production (88).

The observed association between an increased proportion of CD3^+^HLA-DR^+^ cells and reduced rsFC in brain networks potentially provides insight into how chronic inflammation might alter brain function during aging. The MPFC, a part of the default mode network, plays a key role in coordinating neurophysiological, autonomic, and behavioral responses to stress (89, 90). It regulates immune functions by interacting with the autonomic nervous system and the hypothalamic–pituitary–adrenal axis, both of which influence immune processes (91). The amygdala, which is central to processing emotional responses, influences autonomic and neuroendocrine responses and impacts immune regulation. The cerebellum, which controls several regions of the hypothalamus, has been suggested to significantly contribute to neuroimmune interactions (92).

Our within-subject analysis revealed that the rsFC changes associated with *ELOVL2* methylation were anatomically distinct from those associated with T-cell activation. This divergence indicates that while epigenetics and peripheral immunity concurrently track with neurophysiological alterations in this cohort, they do not modulate the same brain networks. Instead, *ELOVL2* methylation and T-cell dynamics may represent parallel pathways of a multi-system response.

Additionally, the metabolites of the gut microbiota contribute to the regulation of *ELOVL2* methylation, impacting immune function and inflammatory states that are closely related to aging (93). The gut microbiota is essential for metabolizing dietary PUFAs and converting them into bioactive compound. These metabolites influence local and systemic inflammation and immune responses, impacting the central nervous system (94). Dysbiosis, or an imbalance in gut bacteria, can increase intestinal permeability, allowing pro-inflammatory molecules to enter circulation and potentially reach the brain, affecting brain networks and elevating stress and anxiety responses.

### Study limitations

A primary limitation of this study is the relatively small sample size, which may limit the statistical power to detect subtler effects, though the core findings achieved significance. Additionally, the cohort is highly homogeneous (predominantly female, monoethnic individuals of Russian descent, free of major comorbidities), and our dataset lacks specific information regarding the participants’ dietary patterns and serum lipid profiles. as well as the serum C-reactive protein (CRP) levels in the recruited cohort.

Because the *ELOVL2* pathway operates directly through omega-3 PUFA availability—a variable largely determined by dietary intake, baseline lipid levels, and genetic backgrounds—our study functions strictly as a controlled proof-of-concept. Without direct metabolic data and a larger, more diverse sample, the observed significant effect sizes may represent an optimized phenotype that could be attenuated in a heterogeneous global population where divergent diets and lipid baselines introduce competing variance (95). Measuring CRP would give important supplementary information about systemic inflammation relative to the CD3^+^HLA-DR^+^ marker. Additional analysis of relationships between *ELOVL2* hypermethylation, reduced omega3-PUFA availability, neurophysiological and immune alterations is necessary to confirm the proposed functional links. Future cross-validation in larger, multi-ethnic cohorts with objective dietary assessments, serum lipid profiling and additional inflammation markers is essential to confirm the validity of these findings.

In conclusion, present study results show that greater age-related *ELOVL2* CpG methylation is associated to prolonged P3 latencies and disrupted fMRI rsFC in essential brain networks including dorsal attention, salience and cerebellar circuits. The *APOE4+* genotype can exacerbate these neurophysiological alterations with aging. These data suggest that *ELOVL2* CpG methylation, by potentially modulating PUFA profiles, may act as a candidate epigenetic factor associated with age-related neurophysiological shifts and consequent changes in cognitive performance. Moreover, an increased proportion of CD3^+^HLA-DR^+^ T cells in peripheral blood — a marker of inflammaging—was associated with *ELOVL2* methylation on the one hand and with decreased resting-state rsFC of brain networks on the other hand, suggesting close links between epigenetic aging, chronic inflammation, and brain dysfunction.

## Data Availability

The datasets presented in this study can be found in online repositories. The names of the repository/repositories and accession number(s) can be found in the article/**Supplementary Material**.

## Supplementary material

Supplementary Figure 1 Flow cytometric data: gating strategy and representative histograms.

Supplementary Figure 2 Correlation of CpG methylation of the *ELOVL2* gene with chronological age in nondemented adults (entire sample) **(A),** and in carriers of the *APOE4-* and *APOE4+* genotypes **(B).**

Supplementary Figure 3 Correlation between the ERP P3 latency and chronological age in the nondemented adults (entire sample) **(A)**, and in carriers and noncarriers of the *APOE4+* genotype **(B).**

Supplementary Figure 4 The pattern of circuits in which fMRI resting-state functional connectivity (rsFC) is associatedwith chronological age in nondemented individuals. Results of regression analysis. Abbreviations are the same as those in **Supplementary Table 1**. The orange lines represent positive associations, and the blue lines represent negative associations.

Supplementary Table 1 ROI-to-ROI fMRI resting-state functional connectivity (rsFC) associated with chronological age in nondemented adults. The abbreviations are the same as those in **Supplementary Figure 3**. p-unc, uncorrected p-values; p-FDR, false discovery rate-adjusted p-values; r, right, l, left; Network Salience RPFC, salience network, rostral prefrontal cortex; Network Dorsal Attention IPS, dorsal attention network, intraparietal sulcus; TOFusC, temporal occipital fusiform cortex; sLOC, lateral occipital cortex superior division; Network Dorsal Attention FEF, dorsal attention network, frontal eye fields; Ver7, vermis 7; pITG, inferior temporal gyrus posterior division; SPL, superior parietal lobule; Cereb6 l, cerebellum 6; pSMG, supramarginal gyrus posterior division.
