# Supplementary figures and images for: Association of the methylation of age-related epigenetic marker ELOVL2 with neurophysiological alterations and immunosenescence during aging and its modulation by the APOE genotype
Source: Front Immunol. 2026 Jul 14;17:1803497. doi: 10.3389/fimmu.2026.1803497 (PMC13407353; doi:10.3389/fimmu.2026.1803497)

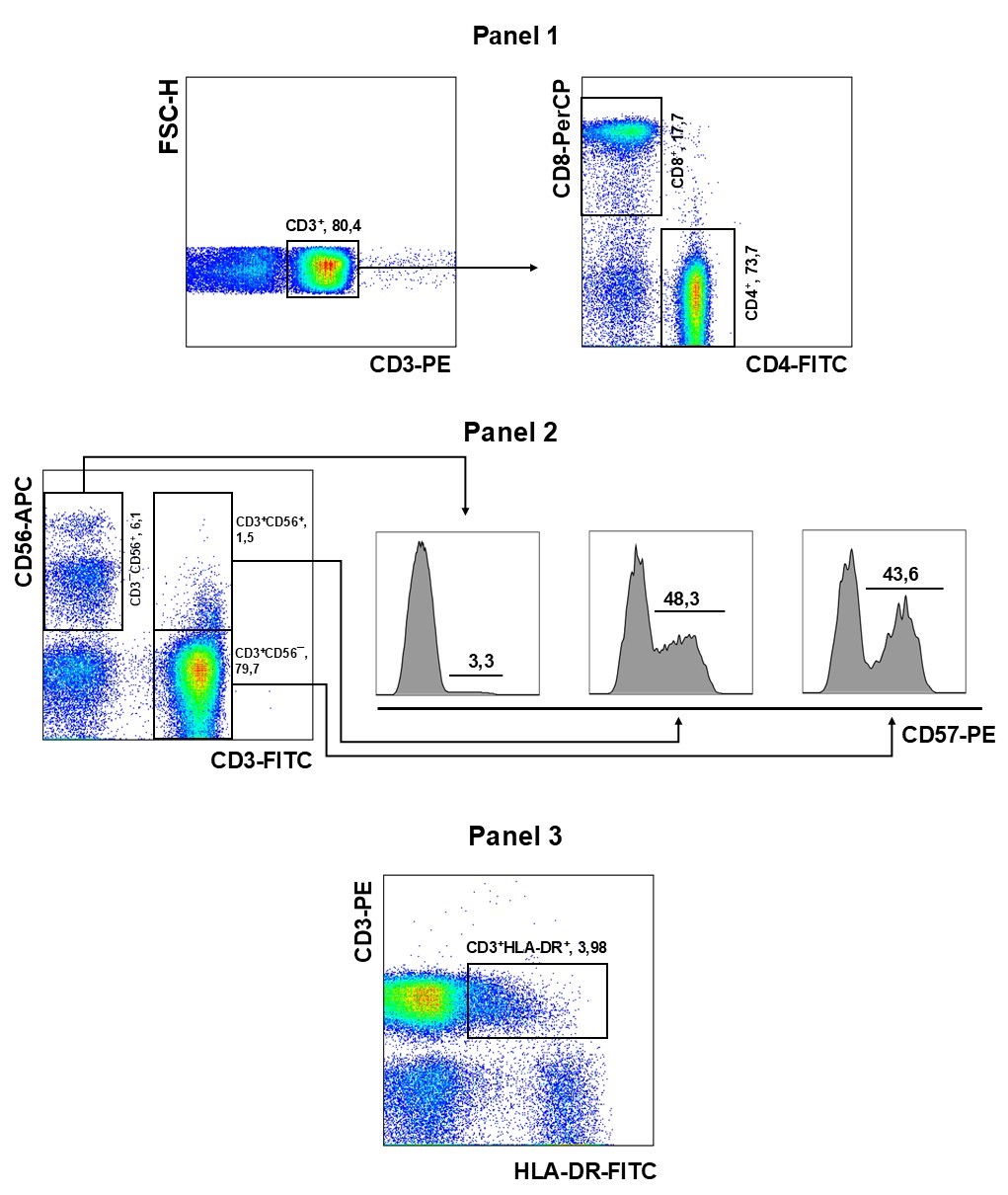

Supplement: Supplementary Figure 1 — Flow cytometric data: gating strategy and representative histograms. [file Image1.jpeg]

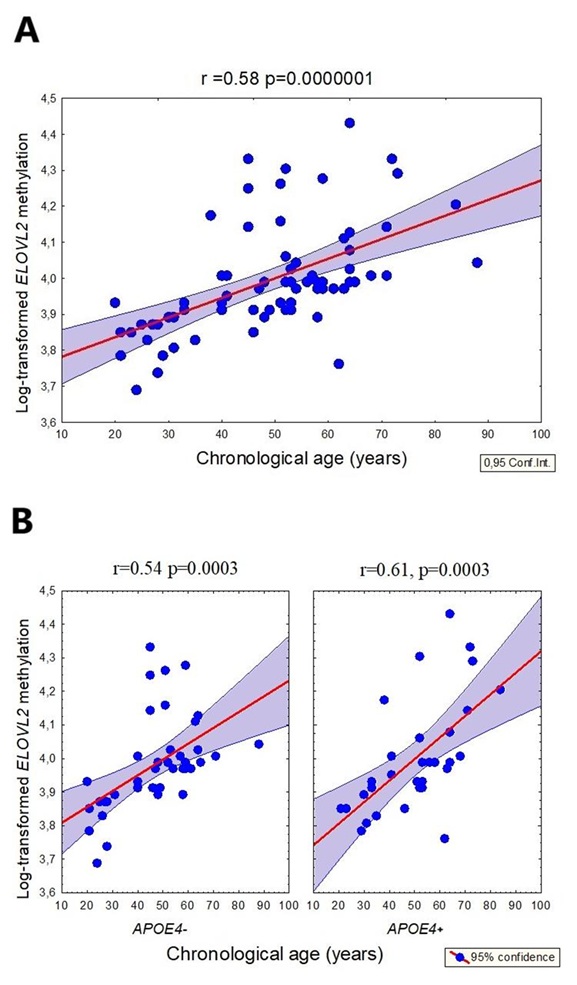

Supplement: Supplementary Figure 3 — Correlation between the ERP P3 latency and chronological age in the nondemented adults (entire sample) (A), and in carriers and noncarriers of the APOE4+ genotype (B). [file Image3.jpeg]

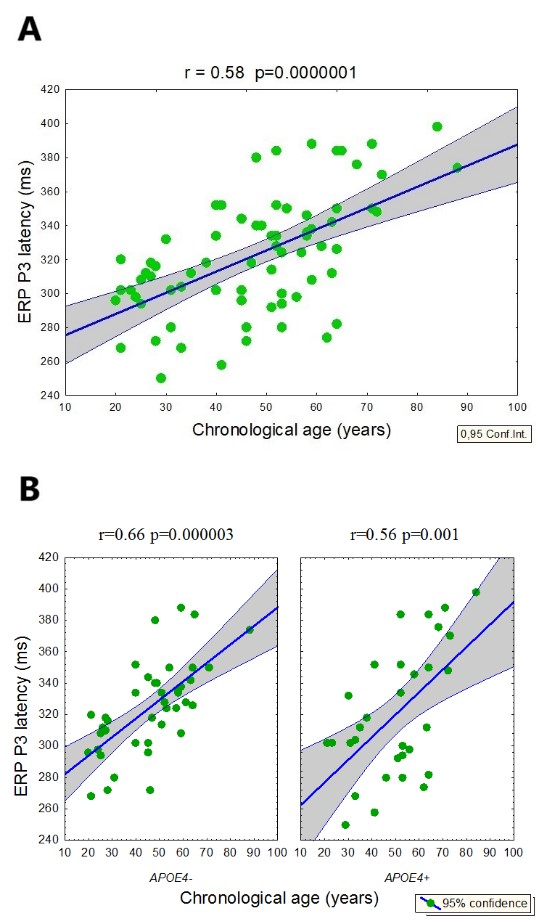

Supplement: Supplementary Figure 4 — The pattern of circuits in which fMRI resting-state functional connectivity (rsFC) is associatedwith chronological age in nondemented individuals. Results of regression analysis. Abbreviations are the same as those in Supplementary Table 1. The orange lines represent positive associations, and the blue lines represent negative associations. [file Image4.jpeg]

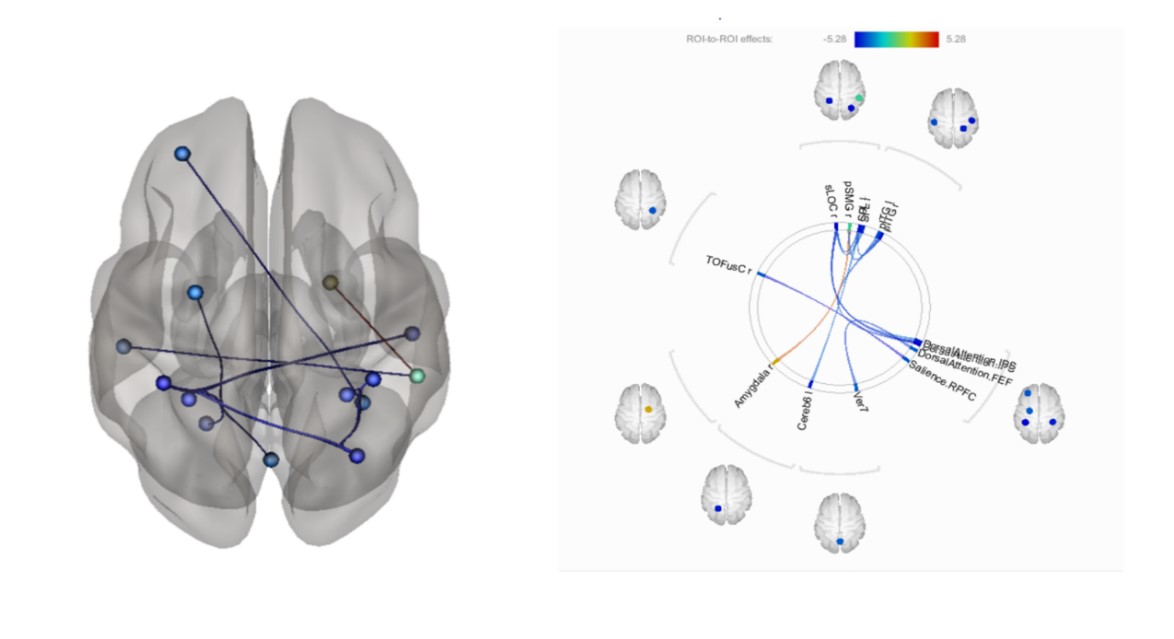

Supplement: Supplementary file 5 [file Image5.jpeg]
