# Supplementary material for: Association of the methylation of age-related epigenetic marker ELOVL2 with neurophysiological alterations and immunosenescence during aging and its modulation by the APOE genotype
Source: Front Immunol. 2026 Jul 14;17:1803497. doi: 10.3389/fimmu.2026.1803497 (PMC13407353; doi:10.3389/fimmu.2026.1803497)
Supplement: Supplementary file 6 [file DataSheet1.zip › Raw_data/Variable list for raw data corresponding to Tables and Figures.docx]

**Variable list for raw data (Excel Format) corresponding to Tables and Figures**

| Figure N | Variables Excel Table |
| --- | --- |
| Figure 1 A | Age (Var 4)  Log-transformed ELOVL2 methylation (Var 6) |
| Figure 1 B | APOE4-(1) vs APOE4+(2) (Var 3)  Age (Var 4)  Log-transformed ELOVL2 methylation (Var 6) |
| Figure 2 A | Age (Var 4)  ERP P3 latency (Var 8) |
| Figure 2B | APOE4-(1) vs APOE4+(2) (Var 3)  Age (Var 4)  ERP P3 latency (Var 8) |
| Figure 3A | Log-transformed ELOVL2 methylation (Var 6)  ERP P3 latency (Var 8) |
| Figure 3B | APOE4-(1) vs APOE4+(2) (Var 3)  Log-transformed ELOVL2 methylation (Var 6)  ERP P3 latency (Var 8) |
| Figure 4 | INDEX Log-transformed ELOVL2 methylation (Var 7)  ERP P3 latency (Var 8) |
| Figure 5 | Age (Var 4)  TOFusC r - Network Salience RPFC L (Var 17)  Network DorsalAttention IPS L -sLOC r (Var 18)  Network DorsalAttention FEF L-Ver7 (Var 19)  pITG r -SPL l (Var 20)  Network DorsalAttention IPS L -pITG r (Var 21)  sLOC r -SPL l (Var 22)  sLOC r -SPL r (Var 23)  SPL l -Cereb6 l (Var 24)  pSMG r -Amygd (Var ala r (Var 25)  pSMG r -pITG l (Var 26)  sLOC r - Network DorsalAttention IPS R (Var 27) |
| Figure 6 | Log-transformed ELOVL2 methylation (Var 6)  pMTG r -aITG r (Var 28)  aITG r - Network Language pSTG r (Var 29)  SPL l -PostCG r (Var 30)  PaCiG r - Network Cerebellar Anterior (Var 31)  PaCiG r -Cereb2 l (Var 32)  PaCiG r -Cereb1 l (Var 33)  PaCiG r -Cereb7 l (Var 34)  SFG r -IFG tri r (Var 35)  SFG r -FOrb r (Var 36)  SFG r -Cereb2 l (Var 37)  PostCG r - Network DorsalAttention IPS L (var 38)  SFG r - Network DorsalAttention FEF L (Var 39)  Network Cerebellar Anterior -PaCiG l (Var 40)  PaCiG r - Network FrontoParietal LPFC R (Var 41)  PaCiG r -IFG tri r (Vaar 42)  SPL l -IC l (Var 43)  Caudate r- Network FrontoParietal LPFC R (Var 44)  Caudate r-FP r (Var 45)  Caudate l - Network VisualOccipital (Var 46) |
| Figure 7 | Log-transformed ELOVL2 methylation (Var 6)  CD3+HLA-DR+(%) (Var 60) |
| Figure 8 | CD3+HLA-DR+(%) (Var 60)  Cereb6 r -Amygdala l (Var 47)  Network DefaultMode MPFC -OP r (Var 48)  Network DefaultMode MPFC-Ver12 (Var 49)  OFusG l -Cereb2 l (Var 50)  Network DefaultMode MPFC - OP l (Var 51)  Cereb2 l -OFusG r (Var 52)  SMA r -TP r (Var 53) |
| **Figure S1** | Flow cytometric data: gating strategy and representative histograms |

| Table 1 | Variables Excel Table |
| --- | --- |
| *APOE4-*  *APOE4+* | APOE4-(1) vs APOE4+(2) (Var 3) |
| Age, yrs | Age (Var 4) |
| Sex m/w | Sex (Var 61) |
| Education, yrs | Education (Var 10) |
| MMSE | MMSE (Var 11) |
| COWAT | COWAT (Var 14) |
| SST | SST errors % (Var 16) |
| Errors on target (%) | Errors on target% (Var 13) |

**Table 2**

The data are the same as those in Table 1, but include only subjects with fMRI data (i.e., variables 17-53).

**Table 3**

The data are the same as those in Table 1, but include only subjects with immunological data (i.e., variables 54-60).

**Table 4.** The data correspond to those in **Figure 5**.

**Table 5.** The data correspond to those in **Figure 6**

**Table 6**

| Subset | Gate |  | Variables Excel Table |
| --- | --- | --- | --- |
| CD3^+^ | Lymphocytes | Lymphocytes | CD3+ (%) (Var 54) |
| CD4^+^CD8^–^ | CD3^+^ | CD3^+^ | CD4+CD8– (%) (Var 55) |
| CD4^–^CD8^+^ | CD3^+^ | CD3^+^ | CD4^–^CD8^+^ (Var 56) |
| CD3^–^CD56^+^ | Lymphocytes | CD3^+^ | CD3^–^CD56^+^ (Var 57) |
| CD57^+^ | CD3^+^CD56^–^ | CD3^+^CD56^–^ | CD57^+^ (Var 58) |
| CD57^+^ | CD3^–^CD56^+^ | CD3^–^CD56^+^ | CD57^+^ (Var 59) |
| CD3^+^HLA-DR^+^ | Lymphocytes | Lymphocytes | CD3^+^HLA-DR^+^(Var 60) |

**Table 7**. The data correspond to those in **Figure 8**
