# Supplementary material for: Association of the methylation of age-related epigenetic marker ELOVL2 with neurophysiological alterations and immunosenescence during aging and its modulation by the APOE genotype
Source: Front Immunol. 2026 Jul 14;17:1803497. doi: 10.3389/fimmu.2026.1803497 (PMC13407353; doi:10.3389/fimmu.2026.1803497)
Supplement: Supplementary Table 1 — ROI-to-ROI fMRI resting-state functional connectivity (rsFC) associated with chronological age in nondemented adults. The abbreviations are the same as those in Supplementary Figure 3. p-unc, uncorrected p-values; p-FDR, false discovery rate-adjusted p-values; r, right, l, left; Network Salience RPFC, salience network, rostral prefrontal cortex; Network Dorsal Attention IPS, dorsal attention network, intraparietal sulcus; TOFusC, temporal occipital fusiform cortex; sLOC, lateral occipital cortex superior division; Network Dorsal Attention FEF, dorsal attention network, frontal eye fields; Ver7, vermis 7; pITG, inferior temporal gyrus posterior division; SPL, superior parietal lobule; Cereb6 l, cerebellum 6; pSMG, supramarginal gyrus posterior division. [file Table1.docx]

**SUPPLEMENTARY MATERIAL**

**Table** S1. ROI-to-ROI fMRI resting-state functional connectivity (rsFC) associated with chronological age in nondemented adults. The abbreviations are the same as those in **Figure 3S**.

| Analysis Unit | T | p-unc | p-FDR |
| --- | --- | --- | --- |
| TOFusC r – Network Salience RPFC L | -5.28 | 0.0000 | 0.0011 |
| Network DorsalAttention IPS L -sLOC r | -5.04 | 0.0000 | 0.0023 |
| Network DorsalAttention FEF L-Ver7 | -4.37 | 0.0001 | 0.0172 |
| pITG r -SPL l | -4.14 | 0.0002 | 0.0185 |
| Network DorsalAttention IPS L -pITG r | -4.11 | 0.0002 | 0.0185 |
| SPL l -pITG r | -4.14 | 0.0002 | 0.0225 |
| sLOC r -SPL l | -4.04 | 0.0003 | 0.0225 |
| sLOC r -SPL r | -3.84 | 0.0005 | 0.0272 |
| SPL l -Cereb6 l | -3.68 | 0.0008 | 0.0422 |
| pSMG r -Amygdala r | 3.87 | 0.0005 | 0.0472 |
| pSMG r -pITG l | -3.78 | 0.0006 | 0.0472 |
| sLOC r – Network DorsalAttention IPS R | -3.52 | 0.0012 | 0.0499 |

Abbreviations: p-unc, uncorrected p-values; p-FDR, false discovery rate-adjusted p-values; r- right, l – left; Network Salience RPFC – salience network, rostral prefrontal cortex; Network Dorsal Attention IPS - dorsal attention network, intraparietal sulcus; TOFusC – temporal occipital fusiform cortex; sLOC – lateral occipital cortex superior division; Network Dorsal Attention FEF - dorsal attention network, frontal eye fields; Ver7 – vermis 7; pITG – inferior temporal gyrus posterior division; SPL - superior parietal lobule;;Cereb6 l - cerebellum 6; pSMG - supramarginal gyrus posterior division.

**Figure S1.** Flow cytometric data: gating strategy and representative histograms

**Figure S2.** Correlation of CpG methylation of the *ELOVL2* gene with chronological age in nondemented adults (entire sample) **(A),** and in carriers of the *APOE4-* and *APOE4+* genotypes **(B)**

**Figure S3.** Correlation between the ERP P3 latency and chronological age in the nondemented adults (entire sample) (**A**), and in carriers and noncarriers of the *APOE4+* genotype **(B)**

**Figure S4.** The pattern of circuits in which fMRI resting-state functional connectivity (rsFC) is associated with chronological age in nondemented individuals. Results of regression analysis. Abbreviations are the same as those in **Table S1**. The orange lines represent positive associations, and the blue lines represent negative associations.
